# Dr. Wm. F. Beck

**Published:** 1916-07

**Authors:** 


**Dr. Wm. F. Beck,** Buffalo 1893, died in Buffalo June 2, aged 59. With the exception of a few years soon after his graduation, he was a practitioner of that city.

